# Pelvic tilt ratio correlates with distinct spinopelvic muscle patterns in adult spinal deformity with sagittal imbalance: a retrospective cohort study

**DOI:** 10.1186/s13018-026-07092-9

**Published:** 2026-07-04

**Authors:** Ronghui Cai, Zhongning Xu, Shuquan Zhang, Jingye Wu, Zhao Lang, Jile Jiang, Yuqing Sun, Tenghui Ge

**Affiliations:** 1 Department of Spine Surgery, Peking University Fourth School of Clinical Medicine, No. 31, Xinjiekou East Street, Xicheng District, Beijing, 100035 People’s Republic of China; 2 Department of Spine Surgery, Beijing Jishuitan Hospital, Capital Medical University, No. 31, Xinjiekou East Street, Xicheng District, Beijing, 100035 People’s Republic of China

**Keywords:** Adult spinal deformity, Pelvic tilt ratio, Sagittal imbalance, Erector spinae, Multifidus, Gluteus medius, Fat infiltration

## Abstract

**Background:**

Pelvic tilt ratio (PTr), defined as pelvic tilt divided by pelvic incidence, may provide an individualized measure of pelvic retroversion in adult spinal deformity (ASD). However, the spinopelvic muscle characteristics associated with different degrees of pelvic retroversion remain unclear. This study aimed to characterize radiographic, clinical, and spinopelvic muscle features stratified by PTr and to identify muscle parameters independently associated with pelvic retroversion in ASD patients with sagittal imbalance.

**Methods:**

Ninety-nine surgically treated ASD patients with preoperative sagittal vertical axis > 50 mm and minimum 2 year follow-up were recruited. Radiographic parameters, patient-reported outcomes, and paraspinal and gluteal muscle parameters were compared across three groups by PTr. LASSO-retained muscle variables, adjusted for age, sex, and body mass index (BMI), were entered into a multivariable linear regression model for PTr.

**Results:**

The Large PTr group showed more severe sagittal malalignment, worse preoperative back pain, and greater postoperative radiographic correction. Higher PTr was associated with smaller cross-sectional areas in all three paraspinal muscles, higher erector spinae fat infiltration (ES FIR), and lower gluteus medius fat infiltration (Gmed FIR). LASSO retained multifidus total cross-sectional area (MF TCSA), ES FIR, and Gmed FIR for the final model. In multivariable linear regression, smaller MF TCSA (B = −3.305, *P* < 0.001), higher ES FIR (B = 0.458, *P* = 0.024), and lower Gmed FIR (B = −0.883, *P* = 0.003) were independently associated with greater PTr.

**Conclusions:**

In ASD patients with sagittal imbalance, higher PTr was associated with more severe deformity, worse preoperative back pain, and a distinct spinopelvic muscle pattern. Smaller MF TCSA, greater ES FIR, and lower Gmed FIR were independently associated with greater PTr.

**Supplementary Information:**

The online version contains supplementary material available at https://doi.org/10.1186/s13018-026-07092-9.

## Background

Adult spinal deformity (ASD) is a heterogeneous disorder that commonly causes pain, disability, postural imbalance, and reduced health-related quality of life [1]. Among the radiographic abnormalities observed in ASD, sagittal malalignment is closely linked to clinical symptoms and treatment strategy [2–4].

Pelvic retroversion represents a key compensatory mechanism in ASD, enabling patients to maintain upright posture and horizontal gaze in the presence of sagittal imbalance [5–7]. Muscle-related characteristics have also been studied as a contributing factor in this compensatory process. Previous studies have demonstrated that paraspinal muscle atrophy and fatty degeneration are closely associated with sagittal malalignment and low back pain [8–10]. In addition, gluteal and lower-limb muscles are also involved in pelvic compensation [11]. However, the combined contributions of paraspinal and gluteal muscles to pelvic compensation remain unclear. Furthermore, given that pelvic incidence (PI) varies substantially across individuals, pelvic tilt (PT) alone may not adequately reflect the extent of pelvic retroversion. Therefore, the pelvic tilt ratio (PTr = PT/PI) has been increasingly adopted as a normalized parameter to better characterize the utilization of pelvic compensatory reserve [12, 13].

This study aimed to investigate the radiographic, clinical, and spinopelvic muscle characteristics across different levels of PTr in ASD with sagittal imbalance, and to identify the specific muscle variables independently associated with PTr.

## Methods

### Study design and patient selection

Consecutive ASD patients with sagittal imbalance attending our hospital for surgery between September 2016 and September 2020 were enrolled. Inclusion criteria were age > 18 years, preoperative standing full-spine radiographs demonstrating sagittal vertical axis (SVA) > 50 mm, minimum 2 year follow-up, and complete baseline and final follow-up radiographic and clinical data. Patients with a history of previous spinal surgery, tumor, infection, trauma, or neuromuscular disease were excluded. This retrospective cohort study was approved by the institutional review board of Beijing Jishuitan Hospital (JL-K2023-367-02), and all study procedures were conducted in accordance with the Declaration of Helsinki with informed consent obtained from all participants.

### Radiographic and clinical assessment

**Fig. 1:**
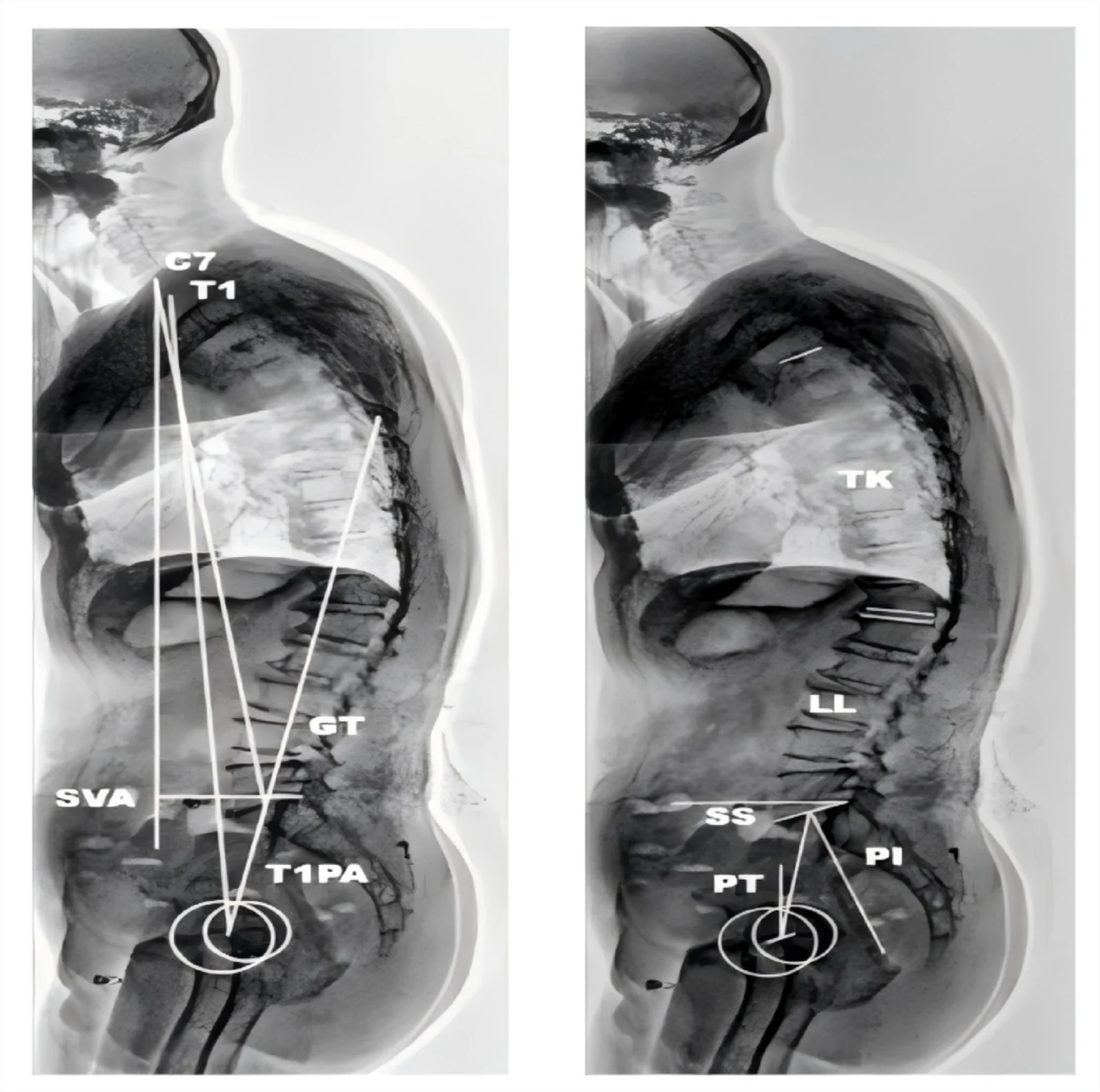
Measurement of sagittal parameters. SVA, sagittal vertical axis; T1PA, T1 pelvic angle; GT, global tilt; PI, pelvic incidence; SS, sacral slope; PT, pelvic tilt; LL, lumbar lordosis; TK, thoracic kyphosis.

Standing full-spine lateral radiographs were obtained preoperatively and at final follow-up. Spinopelvic parameters were measured using Surgimap software (Nemaris Inc., New York, NY, USA), including SVA, T1 pelvic angle (T1PA), global tilt (GT), pelvic incidence (PI), pelvic tilt (PT), sacral slope (SS), lumbar lordosis (LL), thoracic kyphosis (TK), and PI–LL mismatch (Fig. 1). Changes in radiographic parameters above were calculated as postoperative minus preoperative values.

Patient-reported outcome measurements (PROMs) included baseline and final follow-up assessments of the visual analog scale for back pain (VAS-back), visual analog scale for leg pain (VAS-leg), and Oswestry Disability Index (ODI) [14]. Baseline demographic variables included age, sex, body mass index (BMI), symptom duration, and preoperative bone mineral density measured by quantitative computed tomography (CT).

### Paraspinal and gluteal muscle assessment on CT

**Fig. 2:**
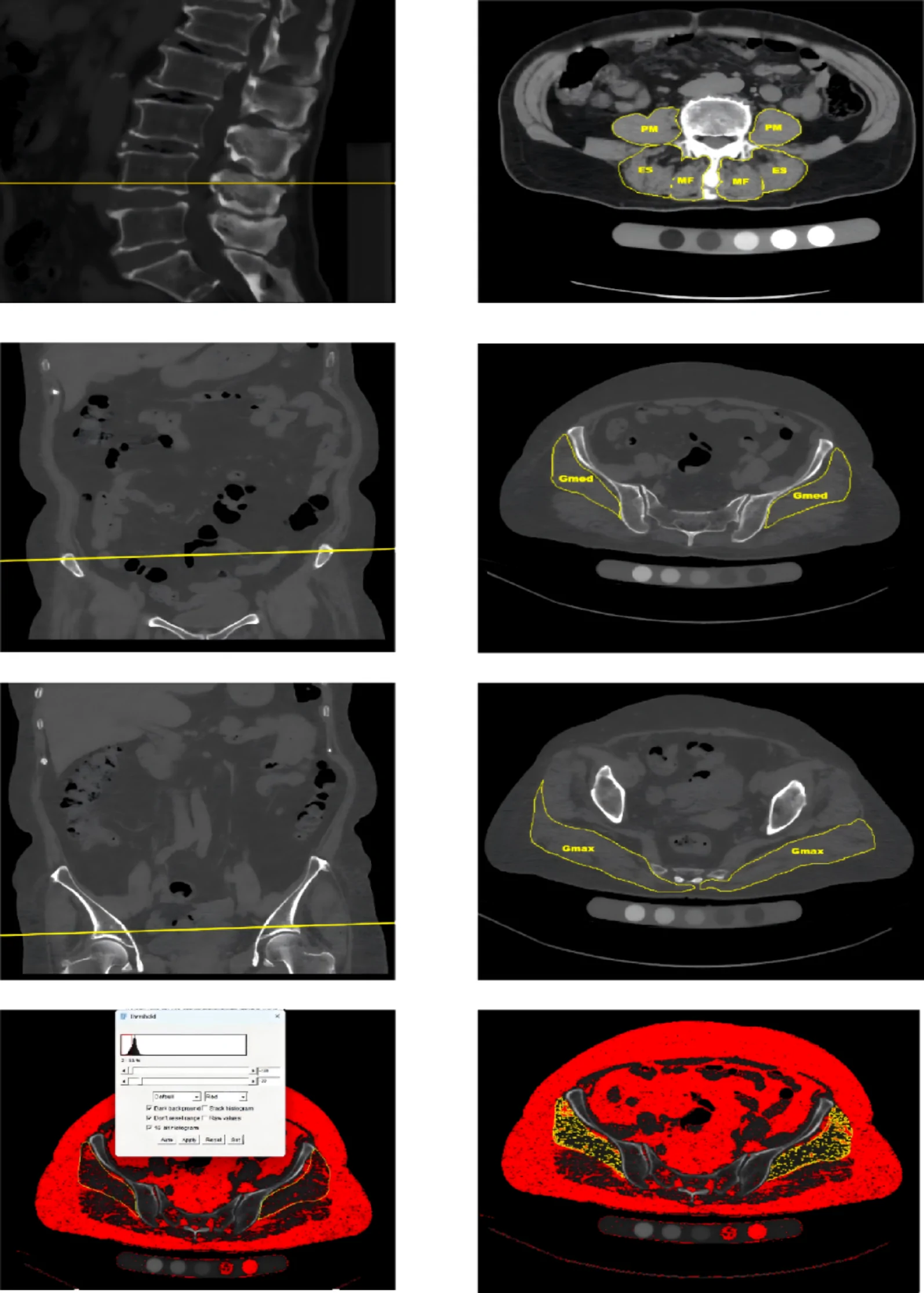
Measurement of paraspinal and gluteal muscles. PM, MF, and ES were measured at the inferior endplate of L4, whereas Gmed and Gmax were measured at the anterior superior iliac spine and superior acetabular margin levels respectively. TCSA was obtained by manual delineation. Fat infiltration was determined using Hounsfield unit thresholds (− 190 to − 30 HU), allowing calculation of FCSA and FIR. Abbreviations: PM, psoas major; MF, multifidus; ES, erector spinae; Gmed, gluteus medius; Gmax, gluteus maximus; TCSA, total cross-sectional area; FCSA, functional cross-sectional area; FIR, fat infiltration rate

Preoperative CT scans were used to assess both paraspinal and gluteal muscles. For paraspinal muscles, axial images at the level of the inferior endplate of L4 were used to evaluate the psoas major (PM), multifidus (MF), and erector spinae (ES). For gluteal muscles, the gluteus medius (Gmed) was measured at the axial level corresponding to the anterior superior iliac spine, and the gluteus maximus (Gmax) at the level of the superior acetabular margin. Regions of interest were manually delineated bilaterally using ImageJ software (version 1.53k, National Institutes of Health, Bethesda, MD, USA) to obtain total cross-sectional area (TCSA). For tissue classification, pixels between − 190 and − 30 Hounsfield units (HU) were defined as fat, whereas pixels between − 29 and + 150 HU were defined as viable muscle tissue [15, 16]. Functional cross-sectional area (FCSA) and fat infiltration rate (FIR) were subsequently calculated based on these predefined thresholds. Representative measurement methodologies for paraspinal and gluteal muscles are shown in Fig. 2.

### Statistical analysis

All statistical analyses were performed using R software (Version 4.5.1; www.r-project.org). Normality was assessed using the Shapiro–Wilk test. Continuous variables are presented as mean ± standard deviation (SD) or median and interquartile range (Q25, Q75), as appropriate. Patients were categorized into tertiles of preoperative PTr using cutoff values of 53.70 and 74.78%. Exploratory intergroup comparisons across ordered PTr categories were performed using one-way analysis of variance with Bonferroni post hoc testing for continuous variables, the Kruskal–Wallis test for non-normally distributed variables, and the chi-square test for categorical variables. Bonferroni correction was applied for post hoc pairwise comparisons, and no additional multiplicity adjustment was performed across all secondary endpoints. Muscle-related variables showing significant intergroup differences were entered into a least absolute shrinkage and selection operator (LASSO) regression model to reduce potential overfitting and collinearity. The remaining variables with non-zero coefficients at the optimal lambda value (determined by 10-fold cross-validation) were further entered into a multivariable linear regression model. The final regression model was performed using complete-case analysis, and patients with missing values in any variable included in the model were excluded. To assess measurement reliability, all radiographic and muscle parameters were independently measured by two observers. Intraclass correlation coefficients (ICCs) were calculated for both interobserver and intraobserver agreement. Statistical significance was defined as a two-sided *P* value < 0.05.

## Results

### Patient stratification and baseline characteristics

A total of 99 ASD patients were included in this study. Baseline demographic and clinical characteristics are summarized in Table 1. There were no significant differences among the groups in age, BMI, symptom duration, and preoperative bone mineral density (BMD). However, a significant difference was observed in sex distribution (*P* = 0.022).

**Table 1:** Baseline demographic and clinical characteristics

Variable	Total (*n* = 99)	Small PTr (*n* = 33)	Middle PTr (*n* = 33)	Large PTr (*n* = 33)	*P* value
Age, y	67.87 ± 7.04	68.52 ± 6.97	67.76 ± 8.87	67.09 ± 5.00	0.630
Sex, Female, *n* (%)	78 (78.79)	21 (63.64)	30 (90.91)	27 (81.82)	0.022*
Body mass index, kg/m^2^	25.29 ± 4.11	25.97 ± 4.93	25.10 ± 3.08	24.82 ± 4.31	0.518
Duration of symptoms, mo^†^	84 (24, 240)	120 (36, 180)	60 (24, 240)	120 (24, 210)	0.556
BMD, mg/cm^3^	82.13 ± 28.64	87.12 ± 30.08	77.09 ± 30.57	81.71 ± 25.83	0.447

Continuous variables are presented as mean ± standard deviation unless otherwise indicated. Categorical variables are presented as frequency and percentage

PTr, pelvic tilt ratio; BMI, body mass index; BMD, bone mineral density; QCT, quantitative computed tomography

^†^Duration of symptoms is presented as median (interquartile range). Duration of spinal symptoms refers to the time from onset of persistent deformity-related spinal symptoms to surgical admission, including back pain, leg pain, neurogenic claudication, postural imbalance, or other deformity-related symptoms. * indicates *P* < 0.05

### Comparative analysis across groups

Preoperative and postoperative radiographic parameters are summarized in Table 2. ICCs for radiographic and muscle measurements ranged from 0.79 to 0.88, indicating acceptable measurement reliability. The Large PTr group demonstrated the most severe baseline sagittal malalignment. PI did not differ significantly across groups. At final follow-up, sagittal alignment improved in all groups. The magnitude of correction was greatest in the Large PTr group. Despite these larger corrections, postoperative spinopelvic parameter mismatch remained significantly different across groups.

**Table 2:** Spinopelvic parameters stratified by PTr

Variable	Total (*n* = 99)	Small PTr (*n* = 33)	Middle PTr (*n* = 33)	Large PTr (*n* = 33)	*P* value	Post hoc
*Preoperative*						
PTr (%)	67.89 ± 26.03	42.10 ± 8.86	63.13 ± 6.13	98.46 ± 16.89	< 0.001	S vs. M***, S vs. L***, M vs. L***
SVA (mm)	117.26 ± 56.88	101.82 ± 33.81	115.27 ± 61.64	134.67 ± 66.35	0.060	
T1PA (°)	35.49 ± 14.97	24.50 ± 7.75	35.74 ± 13.23	46.24 ± 14.41	< 0.001	S vs. M**, S vs. L***, M vs. L**
GT (°)	44.72 ± 17.67	31.96 ± 9.46	44.80 ± 15.68	57.40 ± 16.94	< 0.001	S vs. M**, S vs. L***, M vs. L**
PI (°)	46.46 ± 12.27	48.64 ± 13.13	48.42 ± 10.79	42.32 ± 12.07	0.058	
PT (°)	30.62 ± 11.58	20.59 ± 7.24	30.62 ± 7.67	40.65 ± 9.58	< 0.001	S vs. M***, S vs. L***, M vs. L***
SS (°)	16.07 ± 13.17	28.77 ± 8.98	17.78 ± 4.72	1.67 ± 6.74	< 0.001	S vs. M***, S vs. L***, M vs. L***
LL (°)	6.27 ± 21.57	24.91 ± 14.44	8.18 ± 12.01	-14.28 ± 16.60	< 0.001	S vs. M***, S vs. L***, M vs. L***
PI-LL (°)	40.19 ± 21.22	23.74 ± 13.38	40.25 ± 14.35	56.59 ± 20.92	< 0.001	S vs. M***, S vs. L***, M vs. L***
TK (°)	18.63 ± 16.10	24.12 ± 15.08	20.07 ± 13.90	11.70 ± 17.05	0.005	S vs. L**
*Postoperative*						
SVA (mm)	39.51 ± 38.09	40.90 ± 42.63	39.38 ± 35.85	38.11 ± 37.31	0.976	
T1PA (°)	19.07 ± 8.34	15.96 ± 7.82	21.01 ± 6.51	20.36 ± 9.99	0.115	
GT (°)	25.19 ± 11.03	20.55 ± 9.21	26.84 ± 7.82	28.51 ± 14.29	0.057	
PI (°)	47.13 ± 11.00	47.73 ± 12.26	47.68 ± 9.88	45.45 ± 11.24	0.737	
PT (°)	22.26 ± 9.82	17.03 ± 7.68	23.74 ± 7.26	27.02 ± 12.67	0.001	S vs. M*, S vs. L*
SS (°)	24.99 ± 9.24	30.70 ± 7.39	23.93 ± 7.47	18.91 ± 9.82	< 0.001	S vs. M*, S vs. L*
LL (°)	30.48 ± 12.63	37.63 ± 12.12	29.25 ± 9.42	22.76 ± 12.93	< 0.001	S vs. M*, S vs. L*
PI-LL (°)	17.22 ± 11.40	11.74 ± 8.18	18.45 ± 9.22	22.70 ± 14.97	0.003	S vs. L*
TK (°)	24.37 ± 12.35	30.32 ± 12.06	21.20 ± 9.82	21.29 ± 13.32	0.026	
*Variation (postoperative - preoperative)*						
∆SVA (mm)	−77.75 ± 45.69	−60.92 ± 35.11	−75.89 ± 49.27	−96.56 ± 53.10	0.022	S vs. L*
∆T1PA (°)	−16.42 ± 11.98	−8.54 ± 7.02	−14.73 ± 10.71	−25.88 ± 11.56	< 0.001	S vs. M*, S vs. L***, M vs. L*
∆GT (°)	−19.53 ± 14.11	−11.41 ± 8.44	−17.96 ± 12.63	−28.89 ± 14.21	< 0.001	S vs. M*, S vs. L***, M vs. L*
∆PI (°)	0.67 ± 0.31	−0.91 ± 0.54	−0.74 ± 0.36	3.13 ± 1.73	0.065	
∆PT (°)	−8.36 ± 6.71	−3.56 ± 2.89	−6.88 ± 5.11	−13.63 ± 7.31	< 0.001	S vs. L***, M vs. L**
∆SS (°)	8.92 ± 5.67	1.93 ± 1.76	6.15 ± 4.89	17.24 ± 7.90	< 0.001	S vs. L***, M vs. L***
∆LL (°)	24.21 ± 17.30	12.72 ± 9.08	21.07 ± 9.90	37.04 ± 13.60	< 0.001	S vs. M*, S vs. L***, M vs. L**
∆PI-LL (°)	−22.97 ± 17.00	−12.00 ± 10.31	−21.80 ± 11.51	−33.89 ± 16.89	< 0.001	S vs. M**, S vs. L***, M vs. L*
∆TK (°)	5.74 ± 13.21	6.20 ± 12.41	1.13 ± 8.92	9.59 ± 12.12	0.257	

Data are presented as mean ± standard deviation. ∆ indicates the variation between postoperative and preoperative values.

PTr, pelvic tilt ratio; SVA, sagittal vertical axis; T1PA, T1 pelvic angle; GT, global tilt; PI, pelvic incidence; PT, pelvic tilt; SS, sacral slope; LL, lumbar lordosis; PI-LL, pelvic incidence minus lumbar lordosis mismatch; TK, thoracic kyphosis

*** indicates *P* < 0.001; ** indicates *P* < 0.010; * indicates *P* < 0.050

PROMs are summarized in Table 3. Preoperatively, VAS-back scores differed significantly across groups and were highest in the Large PTr group (Small PTr, 6.0 ± 2.2; Middle PTr, 6.7 ± 2.1; Large PTr, 8.1 ± 1.6; *P* < 0.001), while no significant differences were observed in ODI and VAS-leg scores among the three groups. The final follow-up PROMs did not differ significantly among groups.

**Table 3:** Patient-reported outcomes stratified by PTr

Variable	Total (*n* = 99)	Small PTr (*n* = 33)	Middle PTr (*n* = 33)	Large PTr (*n* = 33)	*P* value	Post hoc
*Preoperative*						
VAS-back	6.9 ± 2.2	6.0 ± 2.2	6.7 ± 2.1	8.1 ± 1.6	< 0.001	S vs. L**, M vs. L*
VAS-leg	5.4 ± 2.5	5.8 ± 2.5	5.4 ± 2.6	5.1 ± 2.3	0.547	
ODI (%)	58.01 ± 12.81	55.67 ± 13.71	56.83 ± 11.51	61.54 ± 12.74	0.143	
*Postoperative*						
VAS-back	2.7 ± 1.1	2.5 ± 1.3	2.9 ± 1.2	2.7 ± 0.8	0.384	
VAS-leg	2.3 ± 1.1	2.4 ± 1.1	2.3 ± 1.0	2.2 ± 0.9	0.828	
ODI (%)	28.28 ± 10.76	28.68 ± 14.10	27.89 ± 10.95	28.26 ± 5.94	0.958	
*Variation (postoperative - preoperative)*						
∆VAS-back	−4.2 ± 2.2	−3.5 ± 2.3	−3.8 ± 2.1	−5.4 ± 1.8	< 0.001	S vs. L**, M vs. L*
∆VAS-leg	−3.1 ± 2.6	−3.4 ± 2.6	−3.1 ± 2.8	−2.9 ± 2.3	0.737	
∆ODI (%)	−29.73 ± 15.70	−26.99 ± 18.41	−28.94 ± 15.48	−33.27 ± 12.46	0.252	

Notes: ∆ indicates the variation between postoperative and preoperative values (postoperative minus preoperative).

PTr, pelvic tilt ratio; ODI, Oswestry Disability Index; VAS-back, visual analog scale for back pain; VAS-leg, visual analog scale for leg pain

* indicates *P* < 0.050; ** indicates *P* < 0.010

The measurements of paraspinal and gluteal muscles are summarized in Table 4. Overall, higher PTr was associated with smaller paraspinal muscle size, greater ES FIR, and lower Gmed FIR. Compared with the Small PTr group, the Large PTr group had smaller PM TCSA and FCSA, smaller MF TCSA and FCSA, and smaller ES TCSA and FCSA. ES FIR was significantly higher in the Large PTr group, whereas PM and MF FIR did not differ significantly. For the gluteal muscles, TCSA and FCSA of both Gmed and Gmax were comparable across groups. However, Gmed FIR was lower in the Large PTr group (8.84 ± 4.12% vs. 15.38 ± 11.49%, *P* = 0.015), whereas Gmax FIR did not differ significantly.

**Table 4:** Paraspinal and gluteal muscle parameters stratified by PTr

Variable	Total (*n* = 99)	Small PTr (*n* = 33)	Middle PTr (*n* = 33)	Large PTr (*n* = 33)	*P* value	Post hoc
*Psoas major (PM)*						
TCSA (cm^2^)	19.73 ± 7.14	21.34 ± 7.62	21.02 ± 8.08	16.88 ± 4.52	0.022	S vs. L*
FCSA (cm^2^)	18.24 ± 6.97	19.75 ± 7.44	19.59 ± 7.96	15.43 ± 4.29	0.02	S vs. L*
FIR (%)	7.80 ± 6.26	7.62 ± 5.89	7.04 ± 7.34	8.71 ± 5.53	0.576	
*Multifidus (MF)*						
TCSA (cm^2^)	13.67 ± 4.66	16.68 ± 4.77	13.99 ± 3.73	10.36 ± 2.99	< 0.001	S vs. M*, S vs. L***, M vs. L**
FCSA (cm^2^)	8.82 ± 3.52	11.15 ± 3.57	8.68 ± 2.85	6.62 ± 2.52	< 0.001	S vs. M**, S vs. L***, M vs. L*
FIR (%)	36.04 ± 11.50	33.16 ± 8.63	38.17 ± 11.34	36.86 ± 13.74	0.211	
*Erector spinae (ES)*						
TCSA (cm^2^)	27.52 ± 10.70	31.57 ± 13.46	29.08 ± 9.83	21.96 ± 4.61	0.001	S vs. L***, M vs. L*
FCSA (cm^2^)	19.08 ± 8.80	23.17 ± 10.29	19.93 ± 8.45	14.18 ± 4.15	< 0.001	S vs. L***, M vs. L*
FIR (%)	31.38 ± 11.36	26.55 ± 8.92	31.77 ± 12.59	35.84 ± 10.69	0.004	S vs. L**
*Gluteus medius (Gmed)*						
TCSA (cm^2^)	54.37 ± 10.77	54.06 ± 10.37	53.82 ± 11.15	55.24 ± 11.12	0.865	
FCSA (cm^2^)	47.56 ± 9.78	45.53 ± 9.48	47.07 ± 10.16	50.20 ± 9.45	0.164	
FIR (%)	12.24 ± 8.98	15.38 ± 11.49	12.28 ± 8.48	8.84 ± 4.12	0.015	S vs. L*
*Gluteus maximus (Gmax)*						
TCSA (cm^2^)	66.49 ± 17.87	72.58 ± 21.35	62.05 ± 15.77	63.57 ± 13.49	0.052	
FCSA (cm^2^)	52.12 ± 13.49	54.62 ± 13.83	50.08 ± 16.41	51.09 ± 9.94	0.417	
FIR (%)	21.17 ± 10.78	23.85 ± 10.74	20.38 ± 12.70	18.87 ± 8.55	0.192	

Data are presented as mean ± standard deviation

PTr, pelvic tilt ratio; TCSA, total cross-sectional area; FCSA, functional cross-sectional area; FIR, fat infiltration rate; S, Small PTr group; M, Middle PTr group; L, Large PTr group

* indicates *P* < 0.050; ** indicates *P* < 0.010; *** indicates *P* < 0.001

### LASSO and multivariable linear regression for PTr

**Fig. 3:**
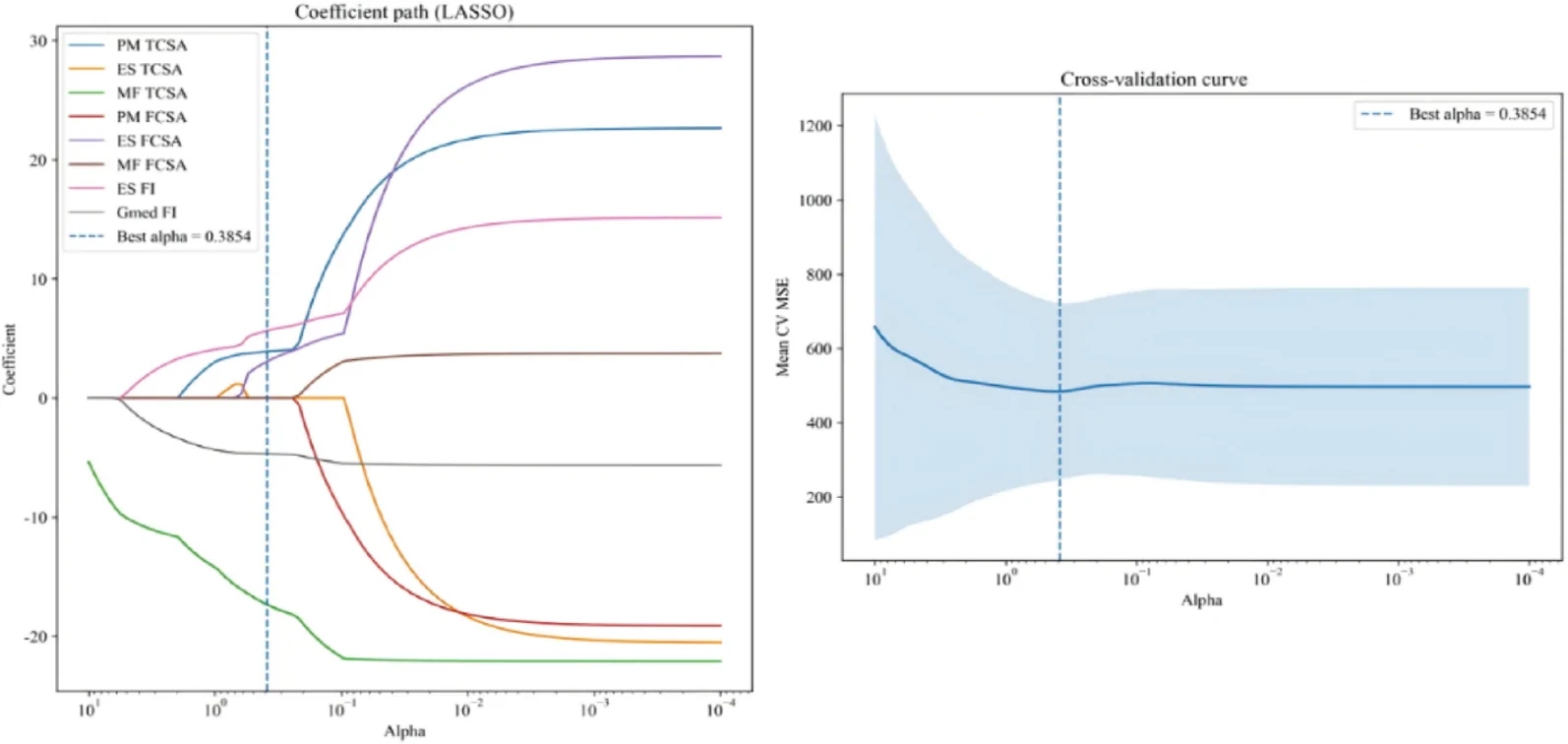
LASSO-based variable selection for muscle parameters. Left, coefficient profiles. Right, 10-fold cross-validation plot for selection of the optimal lambda. LASSO indicates least absolute shrinkage and selection operator

**Fig. 4:**
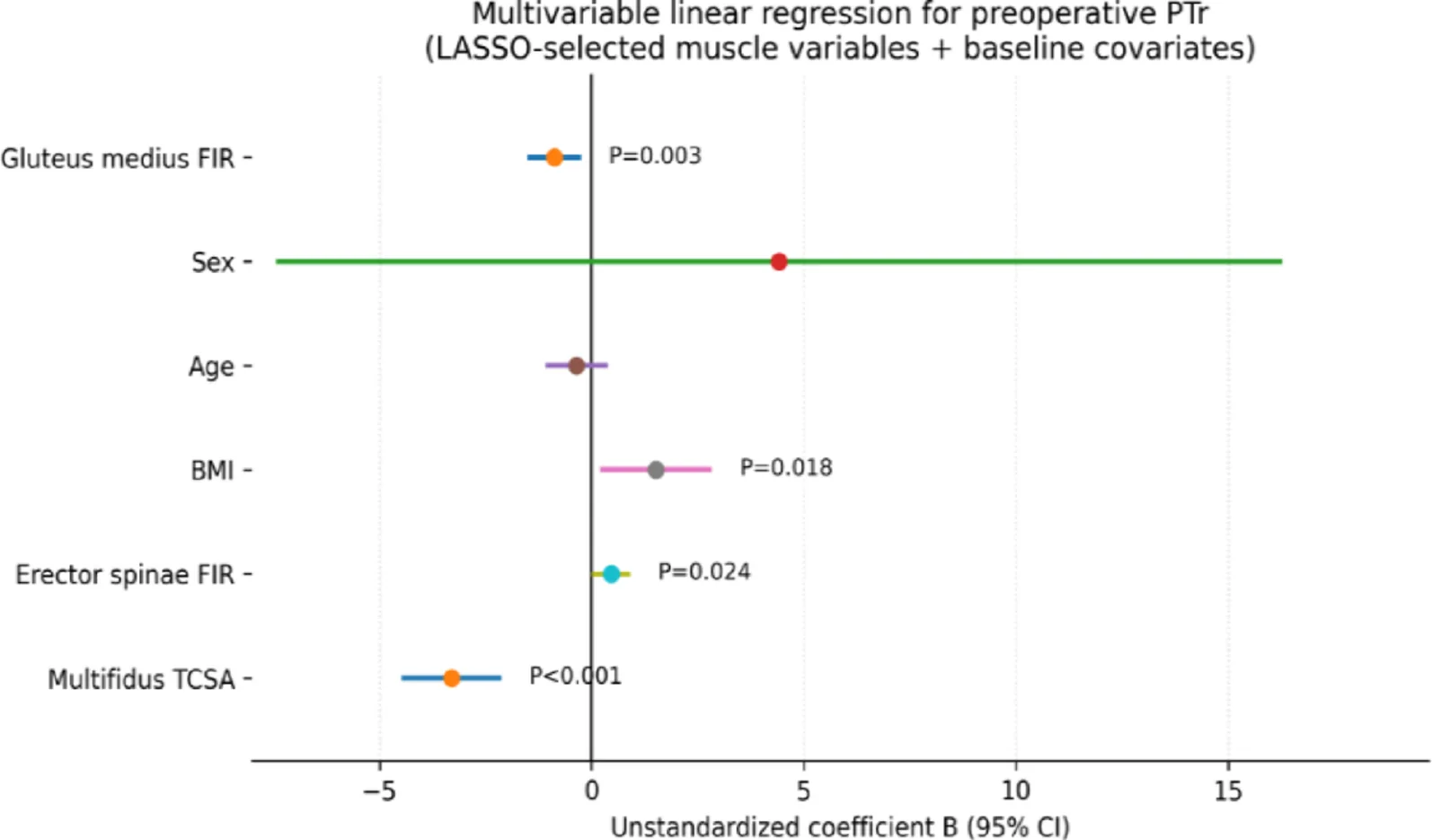
Forest plot of the principal multivariable linear regression model for PTr

Muscle variables showing significant intergroup differences were entered into a LASSO regression model, which identified 5 variables with non-zero coefficients at the optimal lambda value (Fig. 3). All LASSO-selected variables were further evaluated using univariable regression and a full adjusted model including age, sex, and BMI (Supplementary Table S1). Because ES FCSA and ES FIR were derived from the same erector spinae compartment and showed a moderate correlation (*r* = −0.493, *P* < 0.001), ES FIR was retained as the primary ES-related variable because it remained significant in the full adjusted model and was more directly interpretable as a muscle quality parameter. PM TCSA showed coefficient reversal after adjustment for other muscle variables and was not significant in the full adjusted model. Therefore, MF TCSA, ES FIR, and Gmed FIR were retained in the primary reduced model, with additional sequential and sensitivity analyses provided in Supplementary Table S1. The final multivariable model included 86 patients with complete data; details of missingness and included-versus-excluded comparisons are provided in Supplementary Table S2. In the multivariable linear regression model for PTr, MF TCSA (B = −3.305, *P* < 0.001), ES FIR (B = 0.458, *P* = 0.024), and Gmed FIR (B = −0.883, *P* = 0.003) were independently associated with PTr (Table 5; Fig. 4). Bootstrap 95% confidence intervals for the key coefficients were generally consistent with the model-based estimates (Supplementary Table S3). The final model was statistically significant and demonstrated moderate explanatory value (R^2^ = 0.467, *P* < 0.001).

**Table 5:** Multivariable linear regression model for PTr

Variable	B (95% CI)	Standardized *β*	*P* value	VIF
Gluteus medius FIR	−0.883 (−1.459 to −0.308)	−0.283	0.003	1.269
Sex	4.421 (−7.379 to 16.221)	0.066	0.458	1.168
Age	−0.355 (−1.033 to 0.323)	−0.088	0.301	1.064
BMI	1.518 (0.262 to 2.775)	0.234	0.018	1.407
Erector spinae FIR	0.458 (0.061 to 0.855)	0.197	0.024	1.085
Multifidus TCSA	−3.305 (−4.425 to −2.185)	−0.568	< 0.001	1.388

Model fit: *n* = 86; R^2^ = 0.467; adjusted R^2^ = 0.426;* F* = 11.532;* P* < 0.001; Durbin-Watson = 1.429

FIR, fat infiltration rate; TCSA, total cross-sectional area

## Discussion

The principal finding of this study was that PTr was associated with a distinct radiographic, clinical, and muscle profile in ASD patients with sagittal imbalance. Patients in the Large PTr group showed more severe baseline global and regional sagittal malalignment, worse preoperative back pain, and greater absolute radiographic correction after surgery. In addition, muscle parameters showed differences across groups. After LASSO-based variable selection and adjustment for sex, age, and BMI, smaller MF TCSA, higher ES FIR, and lower Gmed FIR remained independently associated with greater PTr. Note that two variables identified by LASSO were excluded during model refinement. ES FCSA and ES FIR provided partly overlapping information from the same erector spinae compartment, and ES FIR was retained because it remained significant in the full adjusted model and more directly represented muscle quality. PM TCSA showed a negative association with PTr in the univariable model but became directionally reversed after adjustment for other muscle variables, suggesting possible suppression effect or shared variance among muscle parameters. Therefore, PM TCSA was not retained in the primary reduced model, although it may still represent a relevant descriptive component of the broader spinopelvic muscle profile.

PTr may provide a more appropriate measure of pelvic compensation than PT alone, as it accounts for individual variability in pelvic morphology. Prior studies have demonstrated that failed pelvic compensation (i.e., low PT/PI despite high SVA) is associated with poorer radiographic and clinical outcomes, whereas higher PTr has been linked to greater postoperative alignment improvement [17, 18]. Consistent with these reports, the present study also showed distinct radiographic and clinical profiles across PTr levels in ASD patients with sagittal imbalance. However, within this imbalanced cohort, PTr tertiles should be interpreted as cohort-specific relative categories rather than diagnostic thresholds for normal or abnormal pelvic compensation. Importantly, our study further examined whether these relative differences in pelvic retroversion were associated with distinct CT-based paraspinal and gluteal muscle morphology.

In this study, the Large PTr group exhibited more severe sagittal deformity and greater compensatory demand. This group was also characterized by reduced paraspinal muscle size and increased ES FIR, suggesting a potential link between muscle degeneration and the extent of pelvic compensation. Notably, the reduced paraspinal TCSA/FCSA in the Large PTr group may be associated with reduced LL, because local sagittal alignment could influence the apparent cross-sectional morphology of paraspinal muscles at the predefined axial CT level. Given that paraspinal muscle quality has been associated with symptom severity and functional impairment [19–21], these findings may partially explain the worse baseline back pain observed in the Large PTr group. Overall, these results provide a basis for further analysis of the combined role of paraspinal and gluteal muscles in pelvic compensation.

Prior studies have linked alterations in spinopelvic and paraspinal muscle morphology to sagittal malalignment and degenerative spinal disorders [10, 22]. Hip- and gluteal-related function has also been implicated in compensation beyond the spine, including dynamic postural control during gait and postoperative alignment recovery [11, 23]. These findings suggest that paraspinal and gluteal muscles may play different roles within the compensatory process: paraspinal muscles appear to be more closely related to structural degeneration associated with sagittal deformity, whereas gluteal muscles may be more relevant to compensation beyond the spine.

**Fig. 5:**
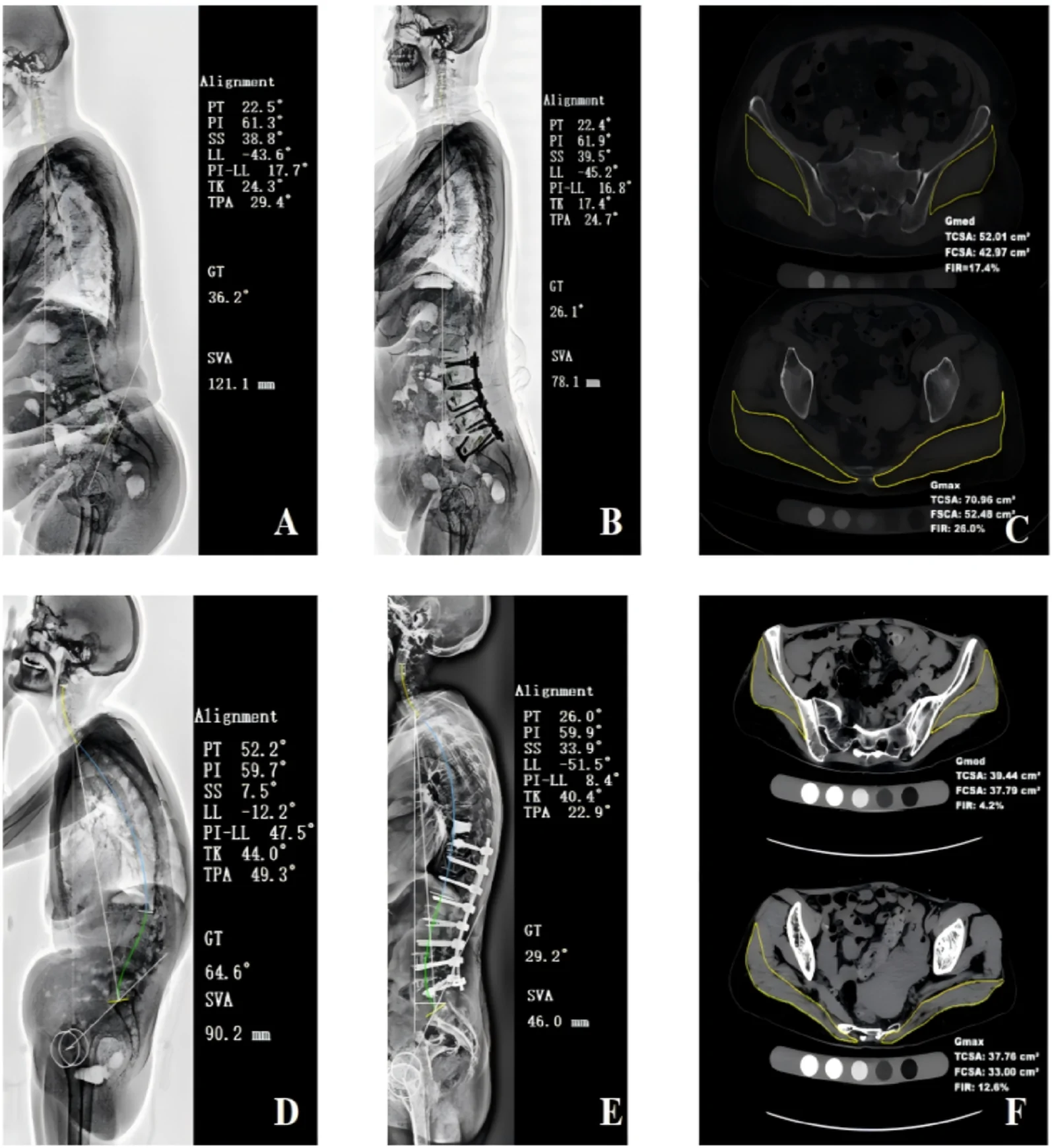
Representative cases from the Small and Large PTr groups. **A**–**C**, A 70-year-old female patient representing the Small PTr group. **A**, Preoperative standing lateral radiograph showing sagittal malalignment with limited pelvic retroversion (SVA = 121.1 mm, PT = 22.5°, PTr = 36.7%). **B**, Postoperative radiograph showed sagittal alignment correction. **C**, Gmed FIR was 17.4% and Gmax FIR was 26.0%** D**–**F**, A 63-year-old female patient representing the Large PTr group. **D**, Preoperative standing lateral radiograph showing sagittal malalignment with marked pelvic retroversion (SVA = 90.2 mm, PT = 52.2°, PTr = 87.4%). **E**, Postoperative radiograph showed sagittal alignment correction. **F**, Gmed FIR was 4.2% and Gmax FIR was 12.6%.

Within this framework, our simultaneous assessment of paraspinal and gluteal muscles suggests that greater PTr is associated with a muscle-specific spinopelvic pattern (Fig. 5). Greater ES FIR and smaller MF TCSA may indicate reduced posterior trunk support for maintaining sagittal alignment, thereby increasing reliance on pelvic compensation. PM TCSA and FCSA were smaller in the Large PTr group without a corresponding increase in PM FIR, suggesting a reduction in cross-sectional area rather than increased fatty infiltration; this finding may also be related to the measurement level, reduced LL or lumbar kyphotic morphology, and local anatomical configuration.

In contrast, the inverse association between Gmed FIR and PTr may suggest relative preservation of gluteal muscle quality in patients with greater compensatory demand, which is consistent with studies linking hip extensor and gluteal-related function to dynamic postural control and sagittal balance in ASD. Lower Gmed FIR may reflect multiple factors, including sex, body habitus, activity level, measurement level, or other unmeasured confounders. Although sex and BMI were adjusted for in the final multivariable model, the female-only analysis and interaction model suggested that this association was not entirely explained by sex composition, but it remains observational and requires further validation (Supplementary Table S4). Unlike Gmed, Gmax parameters did not differ significantly across PTr groups, suggesting that gluteal morphology in relation to pelvic retroversion may be muscle-specific. Given the large size, broad anatomical distribution, and dynamic role of Gmax in hip extension, single-slice supine CT morphology may not fully capture its potential contribution to lumbopelvic compensation.

Clinically, these findings suggest that PTr may help identify patients with a more advanced compensatory state, in whom muscle degeneration is not limited to the lumbar extensors alone but is accompanied by a distinct spinopelvic muscle profile. Such information may be useful for preoperative evaluation and for improving understanding of gluteal muscle changes in ASD. Notably, these findings should be interpreted as morphologic associations, because static CT measurements cannot directly assess dynamic muscle recruitment, gait-related compensation, or load transfer.

Several limitations should be acknowledged. First, this was a retrospective single-center study including only 99 patients, and muscle morphology was assessed at a single preoperative time point; therefore, causal relationships cannot be inferred and generalizability may be limited. Second, CT-based fat quantification provides a static estimate of muscle composition, whereas MRI-based proton density fat fraction is often considered the reference standard for direct assessment of intramuscular fat. Third, the absence of a healthy control group precluded comparison with normal muscle morphology and there is a need to establish an optimal reference value of PTr for defining pelvic compensation. Fourth, objective muscle strength assessment was not performed, and CT-derived muscle morphologic parameters cannot directly reflect muscle functional performance.

## Conclusions

In conclusion, larger preoperative PTr in ASD with sagittal imbalance was associated with more severe sagittal malalignment, worse preoperative back pain, and a distinct spinopelvic muscle pattern characterized by smaller MF TCSA, greater ES FIR, and lower Gmed FIR. These findings suggest that PTr may be useful not only for describing compensatory alignment, but also for characterizing associated muscle morphology in ASD.

## Supplementary Information

Below is the link to the electronic supplementary material.

Supplementary Material 1

## Data Availability

The datasets generated and/or analysed during the current study are not publicly available due to privacy and ethical restrictions but are available from the corresponding author on reasonable request.

## Abbreviations

ASD: Adult spinal deformity
BMD: Bone mineral density
BMI: Body mass index
CI: Confidence interval
CT: Computed tomography
ES: Erector spinae
FCSA: Functional cross-sectional area
FIR: Fat infiltration rate
Gmax: Gluteus maximus
Gmed: Gluteus medius
GT: Global tilt
HU: Hounsfield unit
ICC: Intraclass correlation coefficient
LASSO: Least absolute shrinkage and selection operator
LL: Lumbar lordosis
MF: Multifidus
ODI: Oswestry Disability Index
PI: Pelvic incidence
PI-LL: Pelvic incidence minus lumbar lordosis mismatch
PM: Psoas major
PROMs: Patient-reported outcome measurements
PT: Pelvic tilt
PTr: Pelvic tilt ratio
QCT: Quantitative computed tomography
SD: Standard deviation
SS: Sacral slope
SVA: Sagittal vertical axis
T1PA: T1 pelvic angle
TCSA: Total cross-sectional area
TK: Thoracic kyphosis
VAS-back: Visual analog scale for back pain
VAS-leg: Visual analog scale for leg pain
VIF: Variance inflation factor
