# Supplementary material for: Pelvic tilt ratio correlates with distinct spinopelvic muscle patterns in adult spinal deformity with sagittal imbalance: a retrospective cohort study
Source: J Orthop Surg Res. 2026 Jul 4;21:561. doi: 10.1186/s13018-026-07092-9 (PMC13613695; doi:10.1186/s13018-026-07092-9)
Supplement: Supplementary file 1 [file 13018_2026_7092_MOESM1_ESM.docx]

**Supplementary Tables**

**Supplementary Table S1. Post-LASSO model refinement and sensitivity analyses for LASSO-selected variables**

**Supplementary Table S1A. Model refinement after LASSO-based variable selection.**

| **Variable** | **Standardized LASSO coefficient** | **Univariable B**  **(95% CI)** | **Univariable P value** | **Full adjusted B**  **(95% CI)** | **Full adjusted P value** | **VIF in full model** | **Primary reduced model status** |
| --- | --- | --- | --- | --- | --- | --- | --- |
| PM TCSA | 0.180 | -1.011 (-1.750 to -0.272) | 0.008 | 0.665 (-0.460 to 1.790) | 0.243 | 3.487 | Not retained |
| ES FCSA | 0.102 | -1.212 (-1.781 to -0.643) | < 0.001 | 0.671 (-0.375 to 1.717) | 0.205 | 4.774 | Not retained |
| MF TCSA | -0.669 | -3.319 (-4.269 to -2.370) | < 0.001 | -4.814 (-6.478 to -3.150) | < 0.001 | 3.234 | Retained |
| ES FIR | 0.223 | 0.664 (0.201 to 1.126) | 0.005 | 0.604 (0.120 to 1.088) | 0.015 | 1.703 | Retained |
| Gmed FIR | -0.202 | -1.017 (-1.594 to -0.441) | < 0.001 | -0.854 (-1.422 to -0.285) | 0.004 | 1.309 | Retained |

Notes: The LASSO regression was performed using standardized variables. The full adjusted model included all LASSO-selected variables and covariates age, sex, and BMI.

**Supplementary Table S1B. Sequential models for PM TCSA.**

| **Model** | **Variables included** | **n** | **PM TCSA B**  **(95% CI)** | **P value** | **R²** | **Adjusted R²** |
| --- | --- | --- | --- | --- | --- | --- |
| Model 1 | PM TCSA only | 92 | -1.011 (-1.750 to -0.272) | 0.008 | 0.076 | 0.066 |
| Model 2 | PM TCSA + age + sex + BMI | 92 | -1.059 (-2.008 to -0.110) | 0.029 | 0.096 | 0.054 |
| Model 3 | PM TCSA + retained muscle variables + age + sex + BMI | 86 | 1.051 (0.097 to 2.005) | 0.031 | 0.498 | 0.453 |
| Model 4 | PM TCSA + ES FCSA + retained muscle variables + age + sex + BMI | 86 | 0.665 (-0.460 to 1.790) | 0.243 | 0.508 | 0.457 |

Note: Retained muscle variables were MF TCSA, ES FIR, and Gmed FIR.

**Supplementary Table S1C. Sensitivity models adding ES FCSA and/or PM TCSA to the primary reduced model.**

| Model | Variable | B (95% CI) | P value |
| --- | --- | --- | --- |
| Primary reduced model | Gmed FIR | -0.883 (-1.459 to -0.308) | 0.003 |
|  | ES FIR | 0.458 (0.061 to 0.855) | 0.024 |
|  | MF TCSA | -3.305 (-4.425 to -2.185) | < 0.001 |
| Model adding ES FCSA | Gmed FIR | -0.810 (-1.375 to -0.244) | 0.006 |
|  | ES FIR | 0.713 (0.265 to 1.162) | 0.002 |
|  | MF TCSA | -4.719 (-6.379 to -3.059) | < 0.001 |
|  | ES FCSA | 1.002 (0.116 to 1.887) | 0.027 |
| Model adding PM TCSA | Gmed FIR | -0.914 (-1.477 to -0.351) | 0.002 |
|  | ES FIR | 0.418 (0.029 to 0.808) | 0.036 |
|  | MF TCSA | -4.193 (-5.552 to -2.834) | < 0.001 |
|  | PM TCSA | 1.051 (0.097 to 2.005) | 0.031 |
| Model adding ES FCSA and PM TCSA | Gmed FIR | -0.854 (-1.422 to -0.285) | 0.004 |
|  | ES FIR | 0.604 (0.120 to 1.088) | 0.015 |
|  | MF TCSA | -4.814 (-6.478 to -3.150) | < 0.001 |
|  | ES FCSA | 0.671 (-0.375 to 1.717) | 0.205 |
|  | PM TCSA | 0.665 (-0.460 to 1.790) | 0.243 |

Notes: All sensitivity models were adjusted for age, sex, and BMI. The primary reduced model included Gmed FIR, ES FIR, MF TCSA, age, sex, and BMI. Sex was coded as 0 = female and 1 = male in all regression models. In the model adding both ES FCSA and PM TCSA, neither variable was independently associated with PTr, whereas the three retained variables remained significant.

**Supplementary Table S2. Missing-data pattern and comparison between patients included in and excluded from the final multivariable regression model.**

**Supplementary Table S2A. Missing-data pattern for the final multivariable regression model**

| **Missing-data pattern** | **Gmed FIR** | **ES FIR** | **MF TCSA** | **Number of patients** |
| --- | --- | --- | --- | --- |
| Gmed FIR only | Yes | No | No | 6 |
| ES FIR + MF TCSA | No | Yes | Yes | 5 |
| Gmed FIR + ES FIR + MF TCSA | Yes | Yes | Yes | 2 |
| Total patients excluded |  |  |  | 13 |

Notes: “Yes” indicates missingness for the corresponding variable. Missingness overlapped across variables: Gmed FIR was missing in 8 patients, ES FIR in 7 patients, and MF TCSA in 7 patients. Overall, 13 patients had at least one missing final-model variable and were excluded from the complete-case regression analysis.

**Supplementary Table S2B. Comparison between patients included in and excluded from the final multivariable regression model**

| **Variable** | **Included in final model**  **(n = 86)** | **Excluded from final model**  **(n = 13)** | **P value** |
| --- | --- | --- | --- |
| Age, y | 68.12 ± 6.65 | 66.23 ± 9.36 | 0.496 |
| Female, n (%) | 69 (80.23) | 9 (69.23) | 0.465 |
| BMI, kg/m² | 25.46 ± 4.13 | 24.15 ± 3.91 | 0.279 |
| Duration of symptoms, mo | 120 (28, 180) | 60 (24, 240) | 0.713 |
| PTr (%) | 68.07 ± 26.75 | 66.73 ± 21.49 | 0.842 |
| PTr group, Small/Middle/Large, n | 31/27/28 | 2/6/5 | 0.316 |
| SVA (mm) | 111.71 ± 47.87 | 153.92 ± 92.24 | 0.130 |
| T1PA (°) | 34.30 ± 13.27 | 43.40 ± 22.47 | 0.178 |
| GT (°) | 43.40 ± 15.55 | 53.48 ± 27.22 | 0.215 |
| PI (°) | 45.90 ± 12.31 | 50.18 ± 11.78 | 0.243 |
| PT (°) | 30.13 ± 11.01 | 33.88 ± 14.93 | 0.399 |
| SS (°) | 16.04 ± 13.52 | 16.28 ± 11.07 | 0.944 |
| LL (°) | 7.39 ± 20.78 | -1.15 ± 25.94 | 0.276 |
| PI-LL (°) | 38.51 ± 19.11 | 51.32 ± 30.62 | 0.165 |
| TK (°) | 19.26 ± 14.75 | 14.42 ± 23.53 | 0.483 |

Notes: Continuous variables are presented as mean ± standard deviation, except duration of symptoms, which is presented as median (interquartile range). Categorical variables are presented as frequency or count. P values were calculated using Welch’s t test for continuous variables, the Mann–Whitney U test for duration of symptoms, Fisher’s exact test for sex, and the chi-square test for PTr group distribution.

**Supplementary Table S3. Bootstrap confidence intervals for the final multivariable regression model**

| **Variable** | **B** | **Model-based 95% CI** | **Bootstrap 95% CI** | **Model-based P value** |
| --- | --- | --- | --- | --- |
| Gmed FIR | -0.883 | -1.459 to -0.308 | -1.559 to -0.361 | 0.003 |
| Sex | 4.421 | -7.379 to 16.221 | -7.043 to 18.321 | 0.458 |
| Age | -0.355 | -1.033 to 0.323 | -1.090 to 0.344 | 0.301 |
| BMI | 1.518 | 0.262 to 2.775 | 0.393 to 2.872 | 0.018 |
| ES FIR | 0.458 | 0.061 to 0.855 | 0.032 to 0.942 | 0.024 |
| MF TCSA | -3.305 | -4.425 to -2.185 | -5.532 to -2.195 | < 0.001 |

Notes: The final multivariable regression model was based on complete-case analysis of 86 patients. Sex was coded as 0 = female and 1 = male in regression models. Bootstrap 95% confidence intervals were calculated using 5,000 resamples. P values were derived from the original multivariable linear regression model.

**Supplementary Table S4. Sex-related sensitivity analyses for the association between Gmed FIR and PTr**

**Supplementary Table S4A. Female subgroup baseline and radiographic characteristics across groups.**

| **Variable** | **Small PTr**  **(n = 21)** | **Middle PTr**  **(n = 30)** | **Large PTr**  **(n = 27)** | **P value** |
| --- | --- | --- | --- | --- |
| Age, y | 68.19 ± 5.62 | 67.80 ± 8.72 | 66.44 ± 4.86 | 0.632 |
| BMI, kg/m² | 25.23 ± 2.60 | 25.28 ± 3.17 | 25.69 ± 4.24 | 0.871 |
| Duration of symptoms, mo | 120 (32, 189) | 66 (26, 240) | 120 (24, 240) | 0.966 |
| PTr (%) | 43.79 ± 7.08 | 62.66 ± 6.05 | 99.78 ± 17.47 | < 0.001 |
| SVA (mm) | 98.53 ± 35.52 | 117.39 ± 64.06 | 141.95 ± 68.50 | 0.046 |
| T1PA (°) | 26.40 ± 8.27 | 36.14 ± 13.50 | 47.50 ± 14.58 | < 0.001 |
| GT (°) | 34.77 ± 9.61 | 45.25 ± 15.99 | 59.06 ± 17.12 | < 0.001 |
| PI (°) | 53.90 ± 12.41 | 48.77 ± 10.20 | 42.36 ± 12.40 | 0.004 |
| PT (°) | 23.60 ± 6.42 | 30.68 ± 7.57 | 41.08 ± 9.52 | < 0.001 |
| SS (°) | 31.43 ± 8.77 | 18.07 ± 4.36 | 1.27 ± 6.94 | < 0.001 |
| LL (°) | 28.62 ± 13.27 | 8.79 ± 12.34 | -15.10 ± 17.03 | < 0.001 |
| PI-LL (°) | 25.28 ± 13.16 | 39.98 ± 14.69 | 57.46 ± 20.84 | < 0.001 |
| TK (°) | 23.82 ± 14.33 | 20.10 ± 14.57 | 13.10 ± 16.86 | 0.051 |

**Supplementary Table S4B. Female subgroup muscle morphology characteristics across groups.**

| Variable | Small PTr | Middle PTr | Large PTr | P value |
| --- | --- | --- | --- | --- |
| PM TCSA (cm²) | 17.72 ± 5.82 | 20.28 ± 8.13 | 15.95 ± 3.82 | 0.049 |
| PM FCSA (cm²) | 16.27 ± 5.67 | 18.77 ± 7.92 | 14.50 ± 3.67 | 0.046 |
| PM FIR (%) | 8.20 ± 5.36 | 7.50 ± 7.58 | 9.13 ± 5.87 | 0.660 |
| MF TCSA (cm²) | 15.88 ± 4.40 | 13.71 ± 3.79 | 10.11 ± 3.05 | < 0.001 |
| MF FCSA (cm²) | 10.35 ± 3.37 | 8.41 ± 2.88 | 6.46 ± 2.63 | < 0.001 |
| MF FIR (%) | 34.96 ± 8.28 | 38.76 ± 11.68 | 36.92 ± 14.97 | 0.561 |
| ES TCSA (cm²) | 29.06 ± 14.84 | 28.87 ± 10.34 | 21.14 ± 4.34 | 0.013 |
| ES FCSA (cm²) | 21.38 ± 11.51 | 19.75 ± 8.75 | 13.71 ± 4.18 | 0.007 |
| ES FIR (%) | 26.62 ± 8.71 | 31.84 ± 12.69 | 35.65 ± 11.56 | 0.031 |
| Gmed TCSA (cm²) | 50.62 ± 5.89 | 54.45 ± 9.92 | 55.57 ± 10.84 | 0.183 |
| Gmed FCSA (cm²) | 44.36 ± 6.54 | 47.36 ± 9.16 | 50.37 ± 8.77 | 0.058 |
| Gmed FIR (%) | 12.36 ± 7.23 | 12.88 ± 8.50 | 9.02 ± 3.71 | 0.102 |
| Gmax TCSA (cm²) | 65.64 ± 14.32 | 61.11 ± 15.42 | 64.52 ± 14.29 | 0.571 |
| Gmax FCSA (cm²) | 50.82 ± 12.43 | 48.94 ± 15.77 | 51.10 ± 10.10 | 0.831 |
| Gmax FIR (%) | 22.83 ± 9.35 | 20.89 ± 12.73 | 19.90 ± 8.51 | 0.651 |

**Supplementary Table S4C. Sex-related sensitivity models for the association between Gmed FIR and PTr.**

| **Model** | **Variable** | **B (95% CI)** | **P value** |
| --- | --- | --- | --- |
| Female-only multivariable model | Gmed FIR | -1.046 (-1.766 to -0.325) | 0.005 |
|  | Age | -0.460 (-1.213 to 0.292) | 0.226 |
|  | BMI | 1.863 (0.302 to 3.424) | 0.020 |
|  | ES FIR | 0.331 (-0.104 to 0.767) | 0.134 |
|  | MF TCSA | -3.314 (-4.538 to -2.089) | < 0.001 |
| Interaction model | Gmed FIR | -1.022 (-1.730 to -0.314) | 0.005 |
|  | Sex | -0.439 (-19.043 to 18.166) | 0.963 |
|  | Gmed FIR × Sex | 0.369 (-0.721 to 1.460) | 0.502 |
|  | Age | -0.365 (-1.046 to 0.316) | 0.290 |
|  | BMI | 1.398 (0.088 to 2.708) | 0.037 |
|  | ES FIR | 0.458 (0.060 to 0.856) | 0.025 |
|  | MF TCSA | -3.321 (-4.446 to -2.196) | < 0.001 |

Notes: Data are presented as mean ± standard deviation, except duration of symptoms, which is presented as median (interquartile range). P values in Supplementary Tables S4A and S4B are exploratory and were used to describe female subgroup patterns across PTr tertiles; post hoc pairwise comparisons were not performed for this sensitivity table. In the female-only multivariable model, the dependent variable was PTr, and the predictors were Gmed FIR, age, BMI, ES FIR, and MF TCSA. Model fit: n = 69; R² = 0.451; adjusted R² = 0.408; overall P < 0.001. In the interaction model, the dependent variable was PTr, and the predictors were Gmed FIR, sex, Gmed FIR × sex, age, BMI, ES FIR, and MF TCSA. Model fit: n = 86; R² = 0.470; adjusted R² = 0.422; overall P < 0.001. Sex was coded as 0 = female and 1 = male in all regression models.

**Abbreviations**: ASD, adult spinal deformity; BMI, body mass index; CI, confidence interval; ES, erector spinae; FCSA, functional cross-sectional area; FIR, fat infiltration rate; Gmax, gluteus maximus; Gmed, gluteus medius; GT, global tilt; LASSO, least absolute shrinkage and selection operator; LL, lumbar lordosis; MF, multifidus; PI, pelvic incidence; PI-LL, pelvic incidence minus lumbar lordosis; PM, psoas major; PT, pelvic tilt; PTr, pelvic tilt ratio; SS, sacral slope; SVA, sagittal vertical axis; T1PA, T1 pelvic angle; TCSA, total cross-sectional area; TK, thoracic kyphosis; VIF, variance inflation factor.
